# Factors associated with link workers considering leaving their role: a cross-sectional survey

**DOI:** 10.3399/BJGPO.2024.0128

**Published:** 2025-07-02

**Authors:** Stephanie Tierney, Lucy Moore, Debra Westlake, Kamal Mahtani, David Nunan, Kerryn Husk, Shoba Dawson, Jane Smith, Emma Fuller, Lilly Sabir, Pauline Roberts, Obioha Ukoumunne

**Affiliations:** 1 Nuffield Department of Primary Care Health Sciences, University of Oxford, Oxford, UK; 2 Peninsula Medical School, University of Plymouth, Plymouth, UK; 3 School of Medicine and Population Health, University of Sheffield, Sheffield, UK; 4 University of Exeter Medical School, University of Exeter, Exeter, UK; 5 Oxford City Primary Care Services, Oxford, UK; 6 Oxford Health NHS Foundation Trust, Oxford, UK

**Keywords:** link workers, job retention, social prescribing, primary health care, cross-sectional studies

## Abstract

**Background:**

Social prescribing (SP) link workers (LWs) listen to patients’ concerns and difficulties, and connect them to relevant community assets (groups, organisations, or charities) that can help with their non-medical issues (for example, loneliness, debt, housing). LW retention is key to sustaining SP within primary care.

**Aim:**

To examine occupational self-efficacy, job discrepancy, and other factors as potential predictors of LWs’ intentions to leave or remain in their posts.

**Design & setting:**

Cross-sectional survey involving LWs from the United Kingdom.

**Method:**

An online questionnaire was distributed via SP-related organisations. Questions were on the following: (a) intention to leave the role; (b) demographics; and (c) role experience, including occupational self-efficacy and discrepancy between expectations and reality of the job. Questions were mainly closed, although some allowed LWs to provide a written response. Logistic regression models were fitted to identify predictors, and content analysis used to categorise open-ended responses.

**Results:**

In total, 342 questionnaire responses were included in the analysis. Higher job discrepancy was associated with past (odds ratio [OR] per 30 unit *increase* = 6.86; 95% confidence interval [CI] = 3.91 to 12.0; *P*<0.001) and future (OR = 4.86; 95% CI = 2.70 to 8.72; *P*<0.001) intentions to leave, while lower occupational self-efficacy was associated only with past intentions to leave (OR per 10 unit *decrease* = 1.91; 95% CI = 1.24 to 2.93; *P* = 0.003).

**Conclusion:**

Findings highlight factors influencing LW retention, offering a foundation for targeted interventions, which could include clearer communication about the role during recruitment, and adjusting job descriptions and support when required.

## How this fits in

Link workers (LWs) are one of the additional roles introduced into primary care in England and are key to the delivery of social prescribing (SP). Turnover of LWs is an area for consideration as it can affect the sustainability of SP in primary care. Our study highlighted factors associated with retention of LWs, including inconsistency between what they expect from the job and what it is like in reality, so-called job discrepancy. Interventions could focus on addressing job discrepancy by considering communication associated with recruitment, amending job descriptions and information provided to patients about SP, and educating GPs about what LWs do.

## Introduction

In England, a national roll-out of social prescribing (SP) link workers (LWs), in 2019, formed part of the Additional Roles Reimbursement Scheme (ARRS);^1^ it provides funding in primary care to improve access by introducing new staff to contribute to the skills mix in this setting.^2–4^ LWs support patients with non-medical issues affecting their health and wellbeing (for example, loneliness, financial worries, or housing problems).^5^ They listen to people’s concerns, seek to understand their priorities, and can support individuals to make changes in their life, often by connecting them to local services, groups, or activities (sometimes called ‘community assets’).

The LW role can vary in terms of location and how SP is delivered. Some LWs in England are employed through primary care to work across one or more GP practices, while others are employed through voluntary, community, and social enterprise (VCSE) organisations.^6^ There is also variation in how many patients LWs are expected to see in a day, the format of these meetings (for example, in-person or via the phone), and whether LWs are invited to multidisciplinary team meetings at practices.^7^

LWs have described how burnout, lack of career progression, and poor supervision affect their job satisfaction and can prompt them to consider leaving their posts.^7,8^ The loss of LWs jeopardises the sustainability of SP; these individuals bring with them, or build up, local knowledge about community assets to which they can connect patients.^9^ Hence, when a LW leaves their post, they take with them a wealth of expertise and connections that their replacement will need time to develop alongside establishing their own networks and local links.

Our previous research on the LW role in primary care^7^ suggested that confidence in the role (occupational self-efficacy) and congruence between their job description and what they actually do (job discrepancy) may be important for retention. Self-efficacy reflects the beliefs someone has about their ability to act in a situation; it can shape how they think, behave, and the effort they mobilise when facing challenges or stressors.^10^ Higher self-efficacy has been reported as protective against stress and burnout in the workplace,^11^ and associated with people more positively assessing their occupational situation.^12,13^ In terms of job discrepancy, LWs have reported how the role sounded much simpler on paper.^14^

### Aim

To examine occupational self-efficacy, job discrepancy, and other factors as potential predictors of LWs’ intentions to leave or remain in their posts.

## Method

### Design

An explanatory sequential mixed-methods study was undertaken, in line with our published protocol,^15^ to address the question: How are occupational self-efficacy and job discrepancy associated with social prescribing link workers’ experiences of, and intention to leave, their role in primary care, and what can be done to support their retention? It started with an online questionnaire (reported here), followed by qualitative interviews with LWs (to be reported separately); the latter will explore in more detail issues related to job discrepancy, confidence in the role, and external factors required to support LWs in primary care.

### Data collection

The online questionnaire (hosted by Jisc Online Surveys) was live between October and November 2023. Key measures were the eight-item Occupational Self-Efficacy Scale^16^ and the 12-item Job Discrepancy Scale.^17^ We also asked responders whether they considered leaving their LW role in the past 6 months or had thoughts about doing so in the next 6 months and, if so, why. Further details about areas covered on the questionnaire are in Supplementary Box S1.

### Sample

LWs across the United Kingdom (UK) were invited to take part in the study via emails sent through relevant organisations (see Supplementary Box S2). On the questionnaire we gave a definition of what we meant by the term LW (see Supplementary Box S3). A target sample size of 192 was calculated to be large enough to detect a mean difference in scores on the Occupational Self-Efficacy Scale of three units (equivalent to half a standard deviation [SD] on the measure)^16^ between participants who had considered leaving their job and those who had not, with 90% power at the (two-sided) 5% level of significance. The calculation assumes that one-third of LWs would have thought about leaving their job, based on previous research.^8^

### Analysis

Main analyses investigated the association of people’s intention to leave their LW role (binary outcome) with occupational self-efficacy^16^ and with job discrepancy.^17^ Additional analyses investigated the association of people considering leaving their role with the following variables identified through our own and other existing work: how they were employed (for example, through primary care, a VCSE organisation, a local council); length of time working as a LW; perceptions of the role as a job or vocation; time available to connect with the VCSE sector; time available to undertake training; supervision provided about patient cases and about their own emotional wellbeing; being part of a peer support group; age; and gender (male, female, or non-binary or other). Logistic regression was used to examine the association between potential predictors and the binary outcome (considered leaving their role). Analyses were adjusted for age. Categorical variables were incorporated in the models by using indicator (dummy) variables. Odds ratios (ORs) for categorical variables indicate the relative increase in the odds of intending to leave for a given category relative to a reference category. All analyses were undertaken using Stata (version 18) software.

Qualitative data collected from responses to open questions were analysed using ‘conventional content analysis’.^18^ This involved inductive category development through initially applying codes to each part of the qualitative data for a specific question, and then clustering codes into a smaller number of broader meaning units. This allowed us to provide a descriptive overview of what was written in response to open-ended questions.

## Results

**Figure 1.**
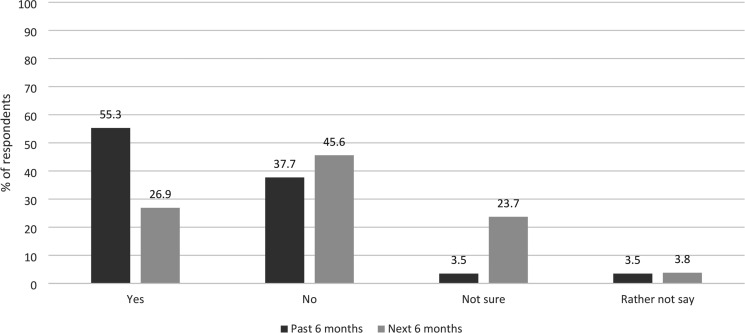
Whether responders were thinking about leaving their role as a link worker (in past 6 months and next 6 months)

We had 348 responses; 342 (98%) were usable, with six excluded because they were not LWs or not based in the UK. As illustrated in Figure 1, over half of responders had considered leaving their role in the past 6 months, whereas just over one-quarter were thinking of doing so in the next 6 months.

### Responder characteristics

As shown in Supplementary Table S4, almost all responders were from England or Scotland, and most self-defined as female (reflecting the gender spilt for this role)^19^ and as White British. The majority were aged ≥40 years (although some younger LWs did respond) and had experienced higher education. One-fifth were managing other LWs. Most responders were employed through a primary care network (PCN) (52.6%) or a VCSE organisation (38.3%). One-third worked part-time. Two-thirds of responders felt they had adequate time in their working week for training, whereas one-quarter had limited time (1 hour a week or less) to make connections with the VCSE sector. We asked about the number of new referrals LWs received each month as an open-ended response; this ranged from 2–150 (the most common number = 20). Some stated they found it hard to provide an answer to this question because of the high frequency of referrals they experienced, having no system to track this, variability in how many referrals they received each month, and working across more than one practice.

### Factors associated with leaving the role

Factors associated with leaving the role are explored in Tables 1 and 2. The mean (SD) occupational self-efficacy score was 41.5 (6.0); a lower score was associated with considering leaving the role in the past 6 months (*P* = 0.003), but not in the next 6 months (*P* = 0.27). The mean difference in self-efficacy score between those who did and those who did not consider leaving in the past 6 months (40.5 versus 42.6, respectively) was less than three, the value considered the minimum important difference and on which the sample size calculation was based. The mean (SD) job discrepancy score was 46.9 (16.4). Greater job discrepancy was associated with thinking about leaving both in the past 6 months and the next 6 months (*P*<0.001 for both analyses).

**Table 1.** Predictors of whether considered leaving post as link worker in past 6 months

Predictor variable	Odds ratio	95% **CI**	*P* value
**Gender**			0.44
Male	reference		
Female	0.77	0.39 to 1.51	
**Age, years**			0.39
21–30	reference		
31–40	0.79	0.32 to 1.91	
41–50	0.58	0.26 to 1.29	
51–60	0.49	0.23 to 1.07	
>60	0.61	0.22 to 1.65	
**Role**			0.13
Primary care network (PCN)	reference		
Voluntary community service	0.83	0.51 to 1.35	
Local authority	1.17	0.28 to 4.93	
Other	0.20	0.05 to 0.78	
**How long have you been link worker?**			0.008
1–6 months	reference		
7–12 months	1.78	0.68 to 4.63	
13–36 months	3.60	1.64 to 7.91	
>36 months	2.22	0.99 to 4.96	
**Do you regard role as job or vocation?**			0.001
Job	reference		
Vocation	0.31	0.15 to 0.64	
**Part of peer support group**			0.008
No	reference		
Yes	0.50	0.30 to 0.84	
**Time to connect with voluntary community sector**			0.008
Up to 1 hour a week	reference		
Two hours/half a day a week	0.51	0.27 to 0.94	
One day a week or more	0.33	0.17 to 0.67	
**Time in working week to undertake training**			<0.001
Disagree	reference		
Neutral	0.36	0.14 to 0.97	
Agree	0.18	0.09 to 0.38	
**Receives supervision about patient cases**			0.02
Never	reference		
Every 2–6 months	0.63	0.27 to 1.51	
Every 4–6 weeks	0.38	0.18 to 0.82	
At least once a fortnight	0.28	0.11 to 0.69	
**Receives supervision about own wellbeing**			0.001
Never	reference		
Every 2–6 months	0.28	0.11 to 0.71	
Every 4–6 weeks	0.19	0.08 to 0.45	
At least once a fortnight	0.15	0.05 to 0.44	
**Occupational self-efficacy (per 10-unit *decrease***)	1.91	1.24 to 2.93	0.003
**Job discrepancy (per 30-unit *increase***)	6.86	3.91 to 12.0	<0.001

Totals range from 290–314.

**Table 2.** Predictors of whether considering leaving post as link worker in next 6 months

Predictor	Odds ratio	95% **CI**	*P* value
**Gender**			0.26
Male	reference		
Female	0.64	0.30 to 1.39	
**Age, years**			0.19
21–30	reference		
31–40	1.64	0.64 to 4.21	
41–50	0.75	0.31 to 1.82	
51–60	0.67	0.28 to 1.60	
>60	0.8	0.26 to 2.47	
**Role**			0.65
Primary care network (PCN)	reference		
Voluntary community service	0.88	0.51 to 1.54	
Local authority	1.32	0.30 to 5.77	
Other	0.39	0.08 to 1.94	
**How long have you been link worker?**			0.01
1–6 months	reference		
7–12 months	2.52	0.77 to 8.21	
13–36 months	3.84	1.41 to 10.4	
>36 months	1.68	0.59 to 4.78	
**Do you regard role as job or vocation?**			0.002
Job	reference		
Vocation	0.35	0.17 to 0.69	
**Part of peer support group**			0.03
No	reference		
Yes	0.52	0.29 to 0.92	
**Time to connect with voluntary community sector**			<0.001
Up to 1 hour a week	reference		
Two hours/half a day a week	0.27	0.14 to 0.54	
One day a week or more	0.24	0.11 to 0.52	
**Time in working week to undertake training**			0.004
Disagree	reference		
Neutral	0.82	0.32 to 2.10	
Agree	0.36	0.18 to 0.69	
**Receives supervision about patient cases**			0.04
Never	reference		
Every 2–6 months	1.31	0.53 to 3.27	
Every 4–6 weeks	0.49	0.21 to 1.13	
At least once a fortnight	0.77	0.29 to 2.08	
**Receives supervision about own wellbeing**			0.02
Never	reference		
Every 2–6 months	0.85	0.36 to 2.02	
Every 4–6 weeks	0.44	0.19 to 1.01	
At least once a fortnight	0.25	0.08 to 0.77	
**Occupational self-efficacy (per 10-unit *decrease***)	1.24	0.82 to 2.06	0.27
**Job discrepancy (per 30-unit *increase***)	4.86	2.70 to 8.72	<0.001

Totals range from 228–247.

Gender, age, and where people were employed (for example, PCN, VCSE sector, or elsewhere) were not associated with thinking about leaving the role. The following were associated with thinking about leaving the role in the past and in the next 6 months:

- **Time in the role:** those in the job for between 1 year and 3 years were most likely to have considered leaving the role (compared with people with less or more time in the job);
- Not being part of a **peer support group**;
- Having limited time in their working week to connect with the **VCSE sector**;
- Lack of time in the working week to undertake **training**;
- Seeing the **role as a job** rather than a vocation;
- Not receiving **frequent supervision** (less than every 2 months) about patient cases or their own wellbeing.

### Qualitative data describing reasons for considering leaving the role

We invited responders who considered leaving their role in the past or next 6 months to describe factors shaping their thinking. Key areas are listed in Supplementary Box S5. Comments in response to a question about what they thought might help to keep LWs in their role are listed in Table 3. Although most people described pressure and feeling overwhelmed by too many referrals, four LWs did write about feeling understimulated and finding the job too slow. In addition, feeling unsafe in the workplace was mentioned by two responders (specific details were not provided, but is something that has also been raised in the interviews we are undertaking for the project).

**Table 3.** Factors participants stated would help to retain link workers in their role

Broad concept	Components	Supporting data
Working conditions	Setting: having a room to see patients in, better IT systems to record patient contacts and to reduce administration burden Support: from managers, including supervision, from peers, receiving appropriate training, emotional wellbeing checks Salary Security: permanent or longer contracts, carer progression opportunities, having enough staff to safely support patients and to have manageable workloads	*‘Higher pay, more training encouraged, more time together as a team as remote lone working can be tough …’* (Responder 22) *‘Stability of the post. 5 to 7 years contract so I can concentrate on the role more and less about whether I will have a job and have to apply for a new one.’* (Responder 42) *‘I think an increase in pay … would make us feel more valued, even though we don’t do it for the money. Having said that, some of the benefits my organisation has offered (eg, carers leave, wellbeing days) have definitely helped me to stay as I feel like my organisation cares about my wellbeing …’* (Responder 46) *‘By being employed by organisations that don’t actually manage us, there is a very disjointed style of support and I do not feel I have a relationship with anyone senior.’* (Responder 62) *‘I know a brilliant social prescriber who left to take another job because it was more money. Would suggest after 6 months of service, when we have developed that local knowledge, that pay should go up.’* (Responder 102) *‘Peer support is vital when we spend so much time lone working.*’ (Responder 153)
Understanding of the role	Clarity around the role so people, including managers, know what it entails, to help with appropriate referrals Recognition as a profession to feel respected and valued by the wider primary care team, and more connected to a practice; might include the ability to acquire a recognised qualification	*‘Medical colleagues using the service for referrals that are ready for change. These roles can be used for putting "heartsink" patients to, after causing anxiety for the referee.’* (Responder 60) *‘… a manager who knows what you are doing, appreciation of complex referrals, less focus on stats, feeling more of a priority, feeling valued.’* (Responder 78) *‘A proper training programme that gives a recognised qualification that is mandatory for the role ...’* (Responder 154) *‘Being valued and understood by other roles. As we are "community" rather than clinical the view from one practice manager was that all we did was go to knitting groups and drink coffee, which is far from the true day-to-day job.’* (Responder 269)
Autonomy	Being able to work in a person-centred way, including where patients are seen Having time to work with patients, to make connections with the VCSE sector, for relevant training, less form filling	*‘So often I have spoken with other people in same/similar roles and surprised how constrained they are. Box-ticking is what seems to happen; the personalised care approach with a holistic modality is not always understood by services...’* (Responder 16) *‘Less form-filling and more patient-focused time …*’ (Responder 104) *‘… a manageable workload so you have time to make community connections or discuss tricky cases with colleagues before acting.’* (Responder 120) *‘… provide proper holistic support rather than the 15 minutes time slots allocated ... which I fight against every day.’* (Responder 125) *‘Being allowed to trust their instincts, being allowed to attend more training and community events, being trusted to manage my own workload.’* (Responder 322)

VCSE = voluntary, community, and social enterprise organisations

Responders were asked whether they saw their role as a job or vocation. Those regarding it as a vocation (82%) described the role as meaningful, allowing them to serve others and/or address social injustice. This meant they accepted low wages and having *‘to sacrifice a lot in personal life’* (responder 30) (due to high caseloads, long working hours, stress). Open comments provided by those regarding their role as a job (18%) suggested their view changed over time, from seeing it as vocational, where they could make a difference, to no longer enjoying it owing to unsupportive managers, excessive workload, uncertain funding, not feeling part of a team, lack of career progression and working with patients they regarded as not taking steps to change their situation. This made them question their ability to endure financial and personal sacrifices.

## Discussion

### Summary

Several factors were associated with LWs considering leaving their role, in both the past and forthcoming 6 months, including greater job discrepancy, perceiving their role as a job rather than a vocation, lack of peer support, and infrequent supervision. Occupational self-efficacy was associated with considering leaving the role in the past 6 months but not the next 6 months. Mean difference in self-efficacy between those who did and did not consider leaving was small at both reference points.

### Strengths and limitations

At the time the questionnaire was completed, there were approximately 3500 LWs across England.^20^ Therefore, responders reflect a proportion of the workforce and may have been motivated to take part because they were thinking about leaving their role; this would have impacted the representativeness of the sample, although not necessarily the estimated relationships between the potential predictors and considering leaving. Given the study’s cross-sectional nature, we caution against interpreting associations as causal. Nevertheless, data have helped us to understand potential reasons why LWs consider leaving their role and how these could be addressed.

### Comparison with existing literature

The percentage of LWs reporting they had considered leaving their role was higher than for other studies on this topic^8^ but fits with recent reports about staff wellbeing in the NHS.^21^ At the point when data were collected (end of 2023) there was uncertainty about whether LWs would continue to be funded through the NHS, highlighting a lack of job security.

Most factors responders mentioned as shaping their decision to leave were extrinsic to the individual LW (for example, supervision provided, time to develop connections with the VCSE sector); factors over which they had little control. This has been noted in other research involving health workers.^22–26^ It might explain why occupational self-efficacy was not as important to turnover; people may feel that more self-efficacy (an intrinsic factor) in conducting their job would not improve their situation as external factors, over which they held little sway, were more influential in shaping their thinking about leaving.

### Implications for research and practice

Results underscore the complexity of retention among LWs and point to potential areas for intervention, such as enhancing vocational identity, educating primary care staff about what they do, providing support during critical tenure periods, and promoting peer support networks. Given the association observed in the data between thoughts about leaving and job discrepancy, aligning job expectations with the realities of the role might help with retention (for example, offering clearer communication during the recruitment process, ongoing support or supervision, and adjusting role descriptions when required).

Longer tenure as a LW was associated with increased odds of considering leaving the role; specifically for LWs employed between 13 months and 36 months. This finding suggests a potential period during which LWs become more likely to contemplate moving on from their job, possibly owing to accumulated stress or unmet expectations. Hence, although attention should be paid to new recruits, and ensuring they feel equipped to undertake the role, those who have been in place for a year or more should not be overlooked; their wellbeing and development needs should be addressed within supervision. Part of this supervision could explore how people describe their role, as our data suggested that defining it as a job rather than a vocation may indicate someone at risk of leaving. Our research underlined the importance of peer support or supervision to improve LW retention. Managers should ensure that these staff have the opportunity to access and engage in such support. They also need protected time to connect to VCSE resources.

It should be noted that findings from this research could have implications for retention of other ARRS roles in primary care (for example, in terms of supervision or support and clarity of what is expected when working in this setting). However, LWs are unique in nature compared with other ARRS roles; they come into primary care lacking the same professional history as, for example, pharmacists, paramedics, or physiotherapists, and are tasked with addressing social rather than medical issues.

While analyses described above were exploratory, they offer insights into the dynamics influencing LWs’ retention and could inform targeted strategies to reduce turnover. Future research could investigate the effectiveness of such interventions; for example, use of independent supervision (from someone outside of the practice[s] where they work), setting up action learning sets or communities of practice, sufficient protected time in the week to work with the VCSE sector. In addition, in-depth research is required around the issues raised in this questionnaire such as feeling unsafe in the workplace. We are in the process of undertaking such research through qualitative interviews with some of the questionnaire responders.

In conclusion, this cross-sectional survey provided valuable insights into factors associated with whether LWs were considering leaving their role. Our findings highlighted that higher job discrepancy was associated with both past and future intentions to leave. Occupational self-efficacy scores were associated only with past intentions. The findings underline the importance of addressing external factors that can affect experiences of this job, and help to ensure that there is greater convergence between what LWs think the position will be like and how it is experienced in reality.

## Data

Data sharing was not part of the consent process with participants, so data cannot be shared.
